# Gender Difference in REM Sleep Behavior Disorder in Japanese Population: Polysomnography and Sleep Questionnaire Study

**DOI:** 10.3390/jcm13030914

**Published:** 2024-02-05

**Authors:** Mamiko Mano, Atsuhiko Nomura, Ryujiro Sasanabe

**Affiliations:** Department of Sleep Medicine and Sleep Disorders Center, Aichi Medical University, Nagakute 480-1195, Japan; nomura.atsuhiko.689@mail.aichi-med-u.ac.jp (A.N.); sasanabe@aichi-med-u.ac.jp (R.S.)

**Keywords:** REM sleep behavior disorder, gender differences, sleep quality, depression, polysomnography

## Abstract

Rapid eye movement (REM) sleep behavior disorder (RBD) is generally common among males and is an established risk factor for neurodegenerative diseases. As the population ages, the prevalence of α-synucleinopathy (such as Parkinson’s disease) is increasing. Additionally, the number of female patients is increasing in Japan. Therefore, we aimed to clarify the clinical characteristics of RBD from the perspective of gender differences in the Japanese population. The proportion of male RBD patients (65.2%) was significantly higher than that of female patients (34.8%). Additionally, female patients (70.5 ± 8.2 years) were significantly older than male patients (67.9 ± 8.0 years, *p* < 0.05). The mean score on the RBD Screening Questionnaire was significantly higher in male patients than in female (8.6 ± 2.9 vs. 7.7 ± 3.1, *p* < 0.05), while the mean score on the Pittsburgh Sleep Quality Index was significantly higher in males (5.9 ± 3.8 vs. 7.2 ± 3.600, *p* < 0.001). The Self-rating Depression Scale in females was 41.7 ± 8.5, and they were more likely to show depressive tendencies than males. In polysomnography, higher rates of obstructive sleep apnea and longer duration of stage N1 sleep were detected in males. After adjusting for age, BMI, and polysomnographic parameters, we found that female RBD patients had significantly worse sleep quality and rates of depression compared to male patients.

## 1. Introduction

Rapid eye movement (REM) sleep behavior disorder (RBD) is a parasomnia characterized by the loss of muscle atonia during REM sleep and abnormal behaviors occurring during REM sleep, often as dream enactments that can cause injury [1,2]. Diagnosis is based on clinical history and polysomnographic documentation of excessive electromyographic activity during REM sleep. RBD may occur alone as RBD or as secondary RBD when comorbid with other medical conditions, such as neurodegenerative diseases, narcolepsy, or psychiatric disorders. The median age of RBD onset is 49 years [3]. RBD in patients aged under 40 years is often associated with narcolepsy. RBD is a well-established risk factor for neurodegenerative diseases such as Parkinson’s disease (PD), Lewy body dementia, and multiple system atrophy [4,5,6]. Prospective studies have estimated that at least 40–65% of patients with RBD will eventually develop a defined neurodegenerative phenotype, especially α-synucleinopathy [7,8,9,10]. A recent study on Japanese patients with RBD reported that 88% of them met the diagnostic criteria for prodromal Parkinson’s disease [11]. PD patients with RBD have more severe symptoms of PD and faster progression than those without RBD [12]. Importantly, RBD is a highly specific marker of future α-synucleinopathy.

The prevalence of RBD in the general population is approximately 0.74–2.01% [13,14,15,16,17]. A male predominance in the presentation of RBD has often been reported with a higher prevalence of RBD in male patients. RBD is clinically more common in male patients. On the other hand, the prevalence of female RBD patients ranges between 13–53.6% of all patients with RBD [14,18,19,20,21].

Although epidemiological studies have reported an equal proportion of males and females with RBD [14,21], case studies often show a male predominance [18,19,20]. Gender differences in the prevalence of RBD may be due to differences in the results between epidemiological studies and case studies. Despite the various studies regarding gender differences in the prevalence of RBD in the general population, this difference remains debatable.

The frequency of RBD complications is not significantly different between male and female PD patients [22]. Furthermore, the neurodegenerative risk in female RBD patients appears to be similar to that in male RBD patients [23]. Despite this imbalance, gender differences of RBD patients are still underreported. Thus, there is a lack of clarity regarding gender differences. PD is more common in men in Europe and the US and it is more common in females in Japan. As the incidence of the disease increases with age, the number of patients is rapidly increasing as the Japanese population ages. Thus, country-specific effects on gender difference in PD may also be found in RBD, an important parameter of α-synucleinopathy prodromal symptoms.

Therefore, this study aimed to evaluate, in detail, the gender differences in Japanese RBD patients using sleep questionnaires (subjective indicators) and polysomnography (PSG) parameters (objective indicators), and to clarify the characteristics of female RBD patients in Japanese population.

## 2. Materials and Methods

### 2.1. Ethical Approval 

The study complied with the principles of the Declaration of Helsinki. The Ethical Committee of Aichi Medical University Hospital approved the study protocol before the collection and analysis of patient data (approval number: 2023-060). Written informed consent was not obtained due to the retrospective nature of this study. Therefore, we disclosed the study protocol on the institutional website (www.aichi-med-u.ac.jp (accessed on 10 August 2023) and offered the potential participants the opportunity to opt out of the study.

### 2.2. Patients

**Figure 1:**
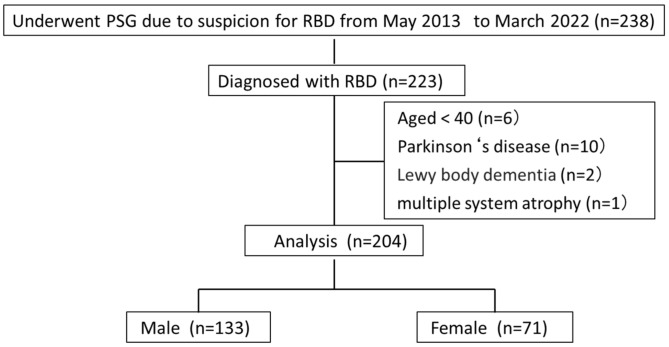
Flowchart of patient inclusion. PSG: polysomnography; RBD: Rapid eye movement (REM) sleep behavior disorder.

We assessed the medical records of patients who had been primarily diagnosed with RBD using video nocturnal polysomnography (v-PSG) at the Department of Sleep Medicine of the Aichi Medical University Hospital from May 2013 to March 2022. A total of 19 patients were excluded: 6 patients aged  <40 years, 10 patients with PD, 2 patients with Lewy body dementia, and 1 patient with multiple system atrophy. Finally, 204 patients (male: *n* = 133, female: *n* = 71) participated in the study (Figure 1).

### 2.3. Sleep Questionnaires

At the first visit, all patients were administered several questionnaires. The Epworth Sleepiness Scale (ESS) questionnaire consists of eight self-rated items, each scored from 0 to 3, which measure a patient’s likelihood of daytime sleepiness in various commonly encountered situations (ESS ≥ 11: excessive daytime sleepiness) [24]. The Pittsburgh Sleep Quality Index (PSQI) comprises 19 self-assessment items that evaluate subjective sleep quality and disability over the preceding month. A higher PSQI score indicates poorer subjective sleep quality (PSQI ≥ 5.5: poor sleep quality) [25]. The Self-rating Depression Scale (SDS) was used to screen patients for the potential presence of depressive disorders. (SDS ≥ 40: depressive states) [26]. Finally, the REM Sleep Behavior Disorder Screening Questionnaire (RBDSQ) was used for the evaluation of RBD symptoms (RBDSQ ≥ 5: suspected RBD) [27].

### 2.4. Polysomnography

V-PSG was performed using an Alice 5 or 6 System (Respironics Inc., Murryville, PA, USA) and a PSG-1100 (Nihon Kohden Co., Tokyo, Japan). The following examinations were performed to continuously monitor biological variables: electroencephalography, bilateral electro-oculography, chin and anterior tibial electromyogramphy, electrocardiography, airflow measurement using a nasal thermistor, measurement of respiratory effort based on thoracic and abdominal movements, body position, snoring sound, and arterial oxygen saturation and a synchronized video system. We used the American Academy of Sleep Medicine (AASM) manual (version 2.6) to score sleep and associated events [14]. The characteristic polysomnographic finding of RBD is REM sleep without atonia (RWA). It is an elevation of motor tone during REM sleep as measured by electromyography (EMG) activity in the chin and/or limb leads. Formal polysomnographic criteria for RWA developed by the AASM require either of the following: sustained elevation of chin EMG activity during REM sleep (higher than 50% of the 30-s epoch duration compared with the minimum amplitude during non-REM sleep) or excessive bursts of transient muscle activity in the chin or limb EMG during REM sleep, defined by the presence of five (50%) or more mini-epochs (30-s epoch is divided into ten sequential 3-s mini-epochs), containing bursts of transient muscle activity. In RBD, excessive transient muscle activity bursts last 0.1 to 5.0 s and are at least four times as high in amplitude as the background EMG activity [28].

### 2.5. Diagnosis of RBD

According to the third edition of the International Classification of Sleep Disorders (ICSD-3), a diagnosis of RBD requires all the following [29]:

- Repeated episodes of sleep-related vocalizations and/or complex motor behaviors.
- These behaviors are documented by polysomnography to occur during REM sleep or, based on the clinical history of dream enactment, are presumed to occur during REM sleep.
- Polysomnographic recording demonstrates REM sleep without atonia (RWA).
- The sleep disturbance is not better explained by another sleep disorder, mental disorder, medication, or substance use.

### 2.6. Statistical Analysis

Comparisons were made between males and females in terms of baseline characteristics, questionnaires, and PSG parameters and were conducted using the Mann–Whitney U test. Fisher’s exact test was conducted for categorical variables. Logistic regression analyses were used to estimate the effects of gender on sleep quality and depression, after adjusting for the age, body mass index (BMI), apnea hypopnea index (AHI), and arousal index. The covariates were selected based on their established clinical relationships with gender difference in RBD. Odds ratios (ORs) are presented, along with the 95% confidence intervals (CIs). All comparisons were two-tailed, and a *p* value < 0.05 was considered statistically significant. All data were analyzed using the SPSS software program version 28 (Armonk, NY, USA: IBM Corp).

## 3. Results

Table 1 shows a comparison between the clinical characteristics of male and female patients with RBD. The proportion of male patients was significantly higher (65.2%) than that of female patients (34.8%). In addition, female patients (70.5 ± 8.2 years) were significantly older than male patients (67.9 ± 8.0 years). Male patients were also significantly larger than female patients, with bigger waist and neck circumferences and higher BMI.

Table 2 shows a comparison of the sleep questionnaire responses between male and female patients with RBD. The average PSQI score was significantly higher in female patients than in male patients (male vs. female: 5.9 ± 3.8 vs. 7.2 ± 3.6, *p* < 0.001). The average SDS score was also significantly higher in female patients than male patients (male vs. female: 38.0 ± 8.7 vs. 41.7 ± 8.5, *p* < 0.001). However, the average RBDSQ score was significantly higher in male patients than in female patients (male vs. female: 8.6 ± 2.9 vs. 7.7 ± 3.1, *p* < 0.05). There was no significant gender difference in ESS scores (male vs. female: 6.7 ± 4.1 vs. 6.5 ± 4.4, *p* = 0.63).

Table 3 shows a detailed comparison of the PSG findings between male and female patients with RBD. Regarding the evaluated PSG parameters, we found several differences between male and female RBD patients. There was no significant gender difference in wake time after onset (WASO) (97.3 ± 57.3 vs. 89.0 ± 57.0, *p* = 0.28). Stage N1 sleep time relative to total sleep time (TST; 48.8 ± 17.7 vs. 36.5 ± 18.2, *p* < 0.001) and stage N2 sleep time relative to TST (32.1 ± 16.4 vs. 45.4 ± 17.4, *p* < 0.001) showed significant gender difference. There was no significant gender difference in stage N3 time relative to TST, but it was higher in females than in males (0.4 ± 1.4 vs. 1.0 ± 2.9, *p* = 0.07). There were no significant differences in REM sleep time relative to TST between male and female patients (18.7 ± 7.7 vs. 17.1 ± 6.6, *p* = 0.17). There were no significant gender differences in sleep latency or REM sleep latency. Male patients also demonstrated a significantly higher apnea/hypopnea index (AHI; 15.1 ± 7.6 vs. 7.2 ± 7.9, *p* < 0.001) and arousal index (29.5 ± 16.3 vs. 22.3 ± 11.8, *p* < 0.05) than female patients. There were no significant differences in oxygen-related items (lowest SpO_2_, CT90) between male and female patients. Proportion of AHI ≥ 5/h was significantly higher in males than in females (68.4 vs. 44.4, *p* < 0.05). Periodic limb movement index (PLMI) was 20.2 ± 28.0. PLMI was significantly higher in females than in males (17.5 ± 26.3 vs. 25.4 ± 30.5, *p* < 0.05).

Table 4 shows the results of the logistic regression analysis for the associations between sleep quality and gender in RBD patients. Female patients were significantly associated with poorer sleep quality in all models, including the unadjusted model (OR = 2.24, 95% CI = 1.235–4.058, *p* < 0.05), in the model adjusted for age (Model 1; OR = 2.19, 95% CI = 1.203–4.001, *p* < 0.05), in the model adjusted for the variables in Model 1 and BMI (Model 2; OR = 2.10, 95% CI = 1.145–3.855, *p* < 0.05), in the model adjusted for the variables in Model 2 and the AHI (Model 3; OR = 2.12, 95% CI = 1.137–3.939, *p* < 0.05), and in the model adjusted for the variables in Model 3 and the arousal index (Model 4; OR = 2.03, 95% CI = 1.082–3.796, *p* < 0.001). Female patients were significantly associated with depression in all models, including the unadjusted model (OR = 1.99, 95% CI = 1.109–3.575, *p* < 0.05), in the model adjusted for age (Model 1; OR = 2.06, 95% CI = 1.135–3.724, *p* < 0.05), in the model adjusted for the variables in Model 1 and BMI (Model 2; OR = 2.16, 95% CI = 1.178–3.954, *p* < 0.05), in the model adjusted for the variables in Model 2 and the AHI (Model 3; OR = 2.38, 95% CI = 1.276–4.437, *p* < 0.001, and in the model adjusted for the variables in Model 3 and the arousal index (Model 4; OR = 2.34, 95% CI = 1.251–4.371, *p* < 0.001).

## 4. Discussion

To our knowledge, this is the first report demonstrating gender differences using PSG parameters and sleep questionnaires in Japanese RBD patients. In this study of patients with RBD, females were significantly older than males at the time of diagnosis, which was consistent with the findings reported by Noboru et al. [23]. The prevalence of RBD is approximately 0.74–2.01% in the general population and approximately 2% in older adults [13,14,15,16]. In our study, the proportion of male patients with RBD was significantly higher (65.2%) than that of female patients (34.8%). Our results are similar to previous reports showing that RBD is a male-dominated parasomnia, with the prevalence in women we observed reporting a prevalence ranging from 13 to 36.5%. This was consistent with previous clinical studies [18,19,20]. Due to the relatively large sample size in our study, the prevalence of female RBD patients may have been distributed more highly among previous clinical case studies. However, our findings were inconsistent with the results of a recent epidemiological study that reported no gender differences in the prevalence of RBD [14,21]. As previously reported, our study is not an epidemiological study, so our study’s finding that RBD is male predominant may be due to sample size effects.

The RBDSQ is a sleep questionnaire for RBD with high sensitivity and specificity, and it is useful as a screening test [27]. In this study, the average score of the RBDSQ was 8.3 ± 3.0, which exceeded the cut-off value. However, female patients with RBD had significantly lower RBDSQ scores compared to male patients. A previous study found that male patients with RBD had a higher proportion of violent dreams and behaviors during sleep [22,30]. The higher RBDSQ scores could reflect the higher rates of violent behavior. In other words, female patients with RBD may have fewer aggressive dreams and behaviors than male patients with RBD, and it appears that clinical history or RBDSQ alone are not as sensitive in diagnosing female patients with RBD, which could lead to their condition being underdiagnosed. Therefore, the prevalence of the observed male predominance in RBD may be biased in clinical studies, because male RBD patients are more likely to exhibit significant motor events and are more likely to seek help in a medical center. There may be more subclinical symptoms among female patients with RBD. Hence, clarifying gender differences in clinical, subjective (sleep questionnaires), and objective (v-PSG) characteristics among patients with RBD is very important.

Previous studies have estimated that at least 40–65% of patients with RBD will eventually develop α-synucleinopathy. Therefore, RBD is a specific marker of future α-synucleinopathy [7,8,9,10]. The comorbidity of sleep disorders (hypersomnia, insomnia, restless legs syndrome (RLS), circadian rhythm dysfunction etc.) in α-synucleinopathy has been reported many times in the past [31]. However, sleep disturbances in patients with RBD have not received significant attention. In our study, the average ESS score in patients with RBD was 6.6, with no significant difference between males and females. Neither male nor female RBD patients exhibited excessive daytime sleepiness. However, the average PSQI score was 6.3 in RBD patients, indicating poor sleep quality, and this score was significantly higher in female patients compared to male patients. Taken together, our results indicate that subjective sleep problems in patients with RBD are related to poor sleep quality rather than to drowsiness, which was more pronounced in females.

Of the patients with RBD, 48.0% had a score of 40 or higher on the SDS, which is indicative of depression; the average SDS score was 39.2 in RBD patients. Female patients with RBD had mild depressive tendencies, with an average SDS score equal or above 40, and significantly higher than that of male patients. Previous studies have shown that 30% of patients with RBD have comorbid depression [31], supporting our finding of high rates of depressive tendencies. Previous studies have reported that depression increases the risk of subsequent PD diagnosis [32]. Therefore, female RBD patients with higher SDS may be at greater risk of progression to neurodegenerative diseases than males.

PSG results in our study showed that 60.3% of RBD patients had comorbid obstructive sleep apnea (OSA). Among patients with RBD, 34–61% have comorbid OSA [33,34,35]; thus, our estimated comorbidity (60.3%) is comparable to those reported in previous studies. Therefore, comorbid OSA is common in patients with RBD. Regarding PSG parameters, patients with RBD demonstrated an AHI of 12.4 per hour, indicating mild OSA. AHI was significantly higher in male than in female patients. The arousal index was also significantly higher in males. However, there was no significant difference between male and female patients with RBD regarding hypoxic burden. This may reflect the protective role conferred by the maintenance of muscle tone during REM sleep. In this study, RBD patients had higher WASO and longer REM latency. There was no difference from the findings presented in past reports [36,37]. Previous reports have not addressed gender differences in sleep architecture in terms of PSG parameters [37]. In our study, objective sleep quality, stage N2 and N3, was significantly lower in males than in females. In contrast, male patients spent more time in stage 1 sleep than female patients did. Objective sleep quality measured using PSG parameters was comparable to that shown in previous results [38]. This difference in objective sleep quality is thought to be related to the gender difference in OSA complication rate.

Depression is commonly reported in patients with OSA [39]. In our study, 48.0% of RBD patients showed a tendency toward depression. RBD with OSA may make patients more prone to depression. Furthermore, in contrast to past studies [35,36], PLMI of female RBD patients was significantly higher than that in male patients in this study. Approximately 80% of restless legs syndrome (RLS) patients exhibit periodic limb movements in sleep. Patients with PD have a high incidence of RLS. It occurs more frequently in females [40]. Therefore, higher PLMI in female RBD patients may be associated with risk factors for progression to neurodegeneration.

To investigate gender differences in sleep quality and depression considering the effects of OSA, we selected age, BMI, AHI, and arousal index as model covariates in the logistic regression analysis since these characteristics have established differential clinical relationships with male and female patients with RBD. The results showed that female RBD patients had poor sleep quality (OR: 2.03–2.24) and higher risk of depression (OR: 1.99–2.34), indicating that they had worse sleep quality than male patients. We found that female patients with RBD suffered more from poor sleep quality and depression than male patients, regardless of the severity of apnea or wakefulness.

Patients with RBD are likely to inflict injuries on their bed partners by aggressive nightmares [41], and the psychological impact and burden in these patients are considerable. Previous research has shown that these patients are more likely to experience depression. In our study, the proportion of patients showing depression was high and tended to be higher among females than males. Therefore, female patients with RBD may experience greater psychological impacts and burdens. Since psychological impacts and burden can reduce sleep quality and increase rates of depression in patients with RBD, poor sleep quality and depression (rather than nightmares) may be the chief complaint in female patients with RBD. Considering that the neurodegenerative risk in female RBD patients is similar to that in male RBD patients, it is recommended to consider a gender perspective when diagnosing RBD.

### Limitations

Our study had several limitations. First, a selection bias was present because the study was conducted at a single center in Japan. Second, we enrolled patients who visited the outpatient clinic with symptoms or were referred by other doctors for suspected RBD. Thus, asymptomatic patients and those with atypical symptoms were excluded. Third, because this was a retrospective study, gender differences in the risk of developing neurological diseases were not investigated.

## 5. Conclusions

The proportion of gender differences in RBD remains unclear. Therefore, female patients with RBD may be underdiagnosed. Female RBD patients had lower RBDSQ than males. Furthermore, contrary to the proportion of stages N2 and N3 in PSG parameters, subjective sleep quality was lower in females than in males. Furthermore, it was confirmed that female RBD patients were more likely to show a tendency toward depression. Until now, insufficient attention has been paid to the influence of RBD on sleep quality and depressive symptoms. It is particularly important to pay attention to sleep quality and depressive symptoms in female patients with RBD.

## Figures and Tables

**Table 1:** Baseline characteristics of the enrolled patients with RBD.

Characteristic	Total (*n* = 204)	Male (*n* = 133)	Female (*n* = 71)	*p* Value
Age (years)	68.8 ± 8.1	67.9 ± 8.0	70.5 ± 8.2	<0.05
BMI (kg/mm^2^)	23.1 ± 2.9	23.5 ± 2.7	22.5 ± 3.3	<0.05
Waist circumference (cm)	87.4 ± 9.6	88.7 ± 9.4	84.9 ± 3.3	<0.05
Neck circumference (cm)	36.2 ± 4.9	38.0 ± 4.8	32.7 ± 2.4	<0.001
Current Smoking (%)	17.2	21.2	9.9	<0.05
Alcohol intake (%)	32.8	39.2	21.2	<0.05

**Table 2:** Comparison of sleep questionnaires between male and female patients with RBD.

Questionnaire	Total (*n* = 204)	Male (*n* = 133)	Female (*n* = 71)	*p* Value
ESS	6.6 ± 4.2	6.7 ± 4.1	6.5 ± 4.4	0.63
PSQI	6.3 ± 3.8	5.9 ± 3.8	7.2 ± 3.6	<0.001
PSQI ≥ 5.5	52.0 (%)	45.1 (%)	64.8 (%)	<0.05
RBDSQ	8.3 ± 3.0	8.6 ± 2.9	7.7 ± 3.1	<0.05
SDS	39.2 ± 8.8	38.0 ± 8.7	41.7 ± 8.5	<0.001
SDS ≥ 40	48.0 (%)	41.4 (%)	59.2 (%)	<0.05

ESS: Epworth Sleep Scale; PSQI: Pittsburgh Sleep Quality Index; RBDSQ: REM Sleep Behavior Disorder Screening Questionnaire; SDS: Self-rating Depression Scale.

**Table 3:** Baseline characteristics and polysomnographic parameters of male and female patients with RBD.

PSG Parameter	Total (*n* = 204)	Male (*n* = 133)	Female (*n* = 71)	*p* Value
Time In Bed (min)	485.4 ± 32.6	485.4 ± 34.0	485.2 ± 29.9	0.79
Total Sleep Time (min)	365.5 ± 64.9	363.9 ± 66.5	368.4 ± 62.1	0.79
WASO (min)	94.5 ± 57.2	97.3 ± 57.3	89.0 ± 57.0	0.28
Sleep Efficiency (%)	75.4 ± 13.2	75.1 ± 13.3	76.2 ± 13.1	0.58
Sleep Latency (min)	17.2 ± 19.2	16.7 ± 20.8	18.27 ± 16.2	0.71
REM Sleep Latency (min)	145.4 ± 88.7	133.5 ± 87.7	167.6 ± 86.9	0.55
REM sleep time/TST (%)	18.1 ± 7.3	18.7 ± 7.7	17.1 ± 6.6	0.17
Stage N1 sleep time/TST (%)	44.6 ± 18.8	48.8 ± 17.7	36.5 ± 18.2	<0.001
Stage N2 sleep time/TST (%)	36.7 ± 17.9	32.1 ± 16.4	45.4 ± 17.4	<0.001
Stage N3 sleep time/TST (%)	0.6 ± 2.1	0.4 ± 1.4	1.0 ± 2.9	0.07
AHI (events/h)	12.4 ± 15.4	15.1 ± 17.6	7.2 ± 7.9	<0.001
AI (events/h)	3.8 ± 9.2	5.4 ± 11.0	0.7 ± 1.8	<0.001
HI (events/h)	8.6 ± 9.2	9.7 ± 10.0	6.5 ± 7.0	<0.05
3%ODI (events/h)	9.6 ± 13.9	11.7 ± 15.8	5.8 ± 7.9	<0.05
Arousal index (events/h)	27.0 ± 15.3	29.5 ± 16.3	22.3 ± 11.8	<0.001
Lowest SpO_2_ (%)	89.9 ± 5.2	89.6 ± 5.4	90.5 ± 4.8	0.23
CT90 (%)	0.76 ± 3.3	0.98 ± 3.9	0.36 ± 1.5	0.19
NREM-AHI (events/h)	12.6 ± 15.9	15.5 ± 18.1	7.1 ± 7.7	<0.001
REM-AHI (events/h)	11.1 ± 16.3	13.0 ± 17.4	7.8 ± 13.7	<0.05
SUP-AHI (events/h)	16.7 ± 19.5	20.8 ± 21.9	9.0 ± 10.1	<0.001
NSUP-AHI (events/h)	4.3 ± 10.0	5.4 ± 11.9	2.1 ± 3.5	0.25
Proportion of AHI ≥ 5 (events/h) (%)	60.3	68.4	44.4	<0.05
Periodic limb movement index (events/h)	20.2 ± 28.0	17.5 ± 26.3	25.4 ± 30.5	<0.05

REM: random eye movement; TST: total sleep time; AHI: apnea hypopnea index; AI: apnea index; HI: hypopnea index; ODI: oxygen desaturation index; SpO2: peripheral capillary oxygen saturation index; CT90: cumulative percentage of time spent at oxygen saturation below 90%; SUP-AHI: AHI in supine position; NSUP-AHI: AHI in non-supine position.

**Table 4:** Associations between sleep quality and gender in patients with RBD.

	Model	OR	95% CI	*p* Value
sleep quality	unadjusted	2.24	1.235–4.058	<0.05
	Model 1	2.19	1.230–4.001	<0.05
	Model 2	2.10	1.145–3.855	<0.05
	Model 3	2.12	1.137–3.939	<0.05
	Model 4	2.03	1.082–3.796	<0.001
depression	unadjusted	1.99	1.109–3.575	<0.05
	Model 1	2.06	1.135–3.724	<0.05
	Model 2	2.16	1.178–3.954	<0.05
	Model 3	2.38	1.276–4.437	<0.001
	Model 4	2.34	1.251–4.371	<0.001

Model 1: adjusted for age. Model 2: adjusted for the parameters in Model 1 + BMI. Model 3: adjusted for the parameters in Model 2 + AHI. Model 4: adjusted for the parameters in Model 3 + ArI. BMI: body mass index; AHI: apnea-hypopnea index; ArI: arousal index; OR: odds ratio; CI: confidence interval.

## Data Availability

Data are contained within the article.
